# Medical assistants’ comic styles and their potential for positive functioning at work: a cross-sectional study including a subgroup analysis

**DOI:** 10.1186/s12875-024-02363-y

**Published:** 2024-05-07

**Authors:** Julia Raecke, René T. Proyer

**Affiliations:** 1 Department ‘Competence Development’, Federal Institute for Vocational Education and Training, Bonn, Germany; 2 Department of Psychology, Martin-Luther-University Halle-Wittenberg, Halle (Saale), Germany

**Keywords:** Primary healthcare, Doctor’s practice, Practice nurse, Ambulant, Coping, Workers’ mental health

## Abstract

**Background:**

Medical assistants are essential for the healthcare system. However, they face several working demands every day, for which they need resources to cope. While several studies show that humour can be a resource for healthcare professionals, studies on humour styles/comic styles in healthcare are scarce. But, as humour styles (e.g., light vs. dark) may have varying – and even negative – effects on positive psychological functioning, it is important to investigate their individual potential for medical assistants. Thus, this study investigates the relationships between medical assistants’ comic styles and their positive psychological functioning at work.

**Methods:**

Applying a cross-sectional design we assessed German medical assistants’ eight comic styles (i.e., benevolent humour, fun, wit, nonsense, irony, satire, sarcasm, cynicism) and facets of positive psychological functioning (e.g., well-being, feeling of competence). We analysed relationships between the variables by means of Pearson correlations, ANCOVAs and hierarchical regressions. All analyses were conducted with the total sample (*N* = 608; completion rate 44%) as well as a large subsample of medical assistants working in general medicine (*N* = 263).

**Results:**

While most of the light styles (e.g., benevolent humour) relate positively to most facets, the dark style *sarcasm* relates negatively. The other dark styles showed coefficients around zero or even slightly positive ones (e.g., satire). Most relationships were also prevalent in the subgroup of medical assistants working in general medicine.

**Conclusions:**

The eight comic styles might have varying potential for medical assistants’ positive psychological functioning at work, with benevolent humour being most adaptive and sarcasm being maladaptive. This study points to the relevance of raising attention regarding the (mal-)adaptiveness of different comic styles of healthcare professionals. Trainings of professionals (e.g., vocational education and training of medical assistants) might integrate the topic of humour (i.e., foster benevolent vs. caution against sarcastic humour) to build and maintain this helpful resource which professionals can use as a tool to master various challenges of everyday work (e.g., cope with stressful situations).

**Supplementary Information:**

The online version contains supplementary material available at 10.1186/s12875-024-02363-y.

## Background

### The medical assistant profession and positive psychological functioning

Medical assistants (MAs) play a crucial role in primary healthcare in several countries [1, 2]. In Germany, working as an MA requires a vocational qualification which is achieved through three-year vocational education and training. This training includes in–company training as well as learning in a vocational school [3]. In the years 2020–2022 over 95% of MA-apprentices in Germany were women [4]. This gender-composition is similar to the internationally more represented nursing profession, which also shows a relatively high share of female apprentices in Germany [5]. However, compared to the nursing profession, which focuses on patient-care, the MA-profession is characterised by a range of multiple different tasks including clinical (e.g., drawing blood, wound care), administrative (e.g., appointment scheduling, ordering practice material) and counselling tasks (e.g., regarding health behaviour of patients) [3]. Therefore, MAs only occasionally work in hospitals but rather in doctors’ practices, where they are an essential support for physicians as well as the first contact person for patients [6]. Despite their important role, MAs – especially due to current societal and health challenges (e.g., pandemic, workforce shortage) – face high working demands (e.g., high workload, low job control) [7, 8]. Though current salary negotiations are progressing well [9], MAs’ incomes are still relatively low [10]. Altogether, these working conditions may lead to poorer well-being or even quitting [8].

One way to counteract these negative outcomes is to increase MAs’ resources. According to the job demands-resources model [11], resources (e.g., social support, optimism) are essential for employees to cope with job demands (e.g., workload) and to increase and maintain several positive employee outcomes (e.g., mental health, work motivation). In this study, in particular, we focus on employee outcomes in terms of MAs’ work-related well-being (e.g., work engagement) and competence (e.g., occupational self-efficacy). Several studies suggest that employees (including healthcare professionals) with high well-being and competence also show better physical health, higher service quality and lower turnover rates [12–14]. For the sake of convenience, we will summarize our study’s outcome variables regarding MAs’ well-being and competence by using the term positive psychological functioning (PPF). PPF is an umbrella term that can be used for a compilation of different constructs that relate to psychological well-being on different levels (e.g., emotionally, cognitive) [14].

### Humour as a resource for PPF and the relevance of humour styles

Humour is a personal resource that can help to maintain one’s PPF in various contexts of life (e.g., at work) [15]. The literature on humour suggests that it is, indeed, a valuable tool for healthcare professionals (e.g., MAs). They use humour for maintaining and/or alleviating their well-being (e.g., reappraise challenging situations with patients, reduce own feelings of stress) [16] as well as to master tasks relevant for their job (e.g., soothe anxious patients, increase team cohesion) [17].

Further, literature also shows that humour manifests in different forms, so-called “styles” (e.g., light vs. dark) and that some styles are maladaptive for one’s and others’ well-being [18]. However, the few studies on humour styles in healthcare are often either non-empirical (i.e., commentaries) or focus on the functions of the styles but not on their relationships to work outcomes (e.g., well-being). Further, they often blend different light and dark styles (e.g., sarcasm, satire and irony) [19], without making a clear distinction. However, as specific light and dark styles can have varying effects on PPF, it is crucial to distinguish between them in this study (e.g., irony and sarcasm are both dark, but only sarcasm aims at belittling) [20]. We examine the relationship between German MAs’ eight distinct comic styles [20] and their PPF; we also test for potential effects of work environments [21] and test the hypotheses for the total sample, but also, separately, for a subsample of MAs working in general medicine, which is the comparatively largest group of MAs in Germany.

#### The light comic styles and their functions for healthcare professionals

Ruch et al.’s comic styles [20] comprise four light and four dark styles. The light styles comprise benevolent humour (i.e., a gentle and forgiving view on weaknesses and mistakes), fun (i.e., good-natured jesting), wit (i.e., clever and witty comments) and nonsense (i.e., liking absurd, illogical humour). Studies on the comic styles show that all four light styles relate positively to well-being and favourable character traits (e.g., life satisfaction, emotional stability) [18, 22]. However, they differ in the strength of these relationships as well as in their functions for health care professionals.

Benevolent humour shows the most robust relationships with positive outcomes [18], and the few studies on the comic styles in healthcare suggest this style to have thoroughly positive functions for everyday work (e.g., facilitating coping with stress, creating supportive social bonds with patients and colleagues) [17, 23]. Further, while fun seems to be especially helpful to improve social relationships (e.g., cohesion in a medical team) [23], wit might be especially helpful to educate patients in a humorous way (e.g., regarding healthy behaviour) [17]. Moreover, nonsense can break the ice with patients and help to distance oneself from severe issues (e.g., illnesses) [24]. Thus, for the light comic styles, we assume the following hypothesis:

##### H1

*The four light comic styles relate positively to MAs’ PPF.*

#### The dark comic styles and their functions for healthcare professionals

The dark comic styles comprise irony (i.e., saying the opposite of what is meant), satire (i.e., criticizing inadequacies with the aim to improve them), sarcasm (i.e., making critical and biting remarks and showing *Schadenfreude*) and cynicism (i.e., a critical, questioning view on morality and hypocrisy). The literature on irony and satire is rather ambivalent. On the one hand, the few studies suggest that a distinct ironic-satiric style goes along with manipulative tendencies and might even lead to intrusive caregiving [25, 26]. On the other hand, authors state that irony and satire can help healthcare professionals to cope with ambiguity or boundaries (e.g., administrative processes, low medical standards) [23]. Further, irony might foster a sense of belonging among healthcare professionals [27], while satire seems to be especially helpful to change others’ amoral attitudes (e.g., of overly demanding patients) [17]. Thus, as the positive functions of irony and satire seem to outweigh the pitfalls, we formulate the following hypothesis taking in mind that light styles likely contribute more to the PPF of MAs than the dark styles:

##### H2

*Irony and satire relate positively to MAs’ PPF.*

Sarcasm and cynicism, on the other hand, both intend to belittle others and go along with maladaptive personality traits (e.g., neuroticism, low agreeableness) [20, 28]. The literature on healthcare professionals’ derogatory humour (i.e., sarcasm and cynicism) suggests both styles to be rather maladaptive. Although some healthcare professionals report finding derogatory humour useful to cope with working demands (e.g., unfairness, emotional stress) [29], they also use it to discredit patients or colleagues who, thus, might feel powerless and anxious [19, 23]. Authors suggest that healthcare professionals, in turn, might develop an emotional distance which can decrease their work motivation and social support from others [19, 30]. Thus, we formulate the following hypothesis:

##### H3

*Sarcasm and cynicism relate negatively to MAs’ PPF.*

### The relevance of context for the adaptivity of comic styles: analysis of a subgroup

MAs work in different medical fields (e.g., paediatrics, gynaecology), which differ regarding certain characteristics, like the typical patient (e.g., children in paediatrics, women in gynaecology) or the severity of diagnoses (e.g., cancer in oncology, broken leg in orthopaedics). As the adaptivity of comic styles may vary regarding such characteristics [21], their adaptability may also vary among medical fields. Medical fields can be roughly divided into “general medicine” and “specific medical fields (e.g., paediatrics, gynaecology)”. In Germany, a high share of MAs works in general medicine [7, 31]. Compared to special fields, the field of general medicine, on the one hand, is quite representative for the sum of all special fields as patients and diagnoses are heterogenous. On the other hand, the field stands out as practices in this field have a high share of regular patients, who are treated and counselled comprehensively over a longer time (i.e., often family practitioner) [32]. We aim to test if the hypothesised relationships between comic styles and PPF are also prevalent in a subgroup of MAs working in general medicine. This should help for a better understanding of the relationships in the field where a high share of MAs works. Therefore, we formulate the following research question:

#### RQ

*Do the assumed relationships between MAs’ comic styles and their PPF also show in a subgroup of MAs working in general medicine?*

## Methods

### Study design and recruitment of participants

We applied a cross-sectional design and conducted an online-survey, programmed with the online-tool on www.soscisurvey.de, version 3.4.16, of the SoSci Survey GmbH (Munich, Germany) [33]. The STROBE Statement − Checklist for cross-sectional studies is used to report this study. We recruited participants via the following channels: (a) MA-related groups on different social media platforms (i.e., Facebook, Instagram), (b) the German nationwide *association of medical occupations* (Verband medizinischer Fachberufe e.V.), and (c) Medical Chambers (Ärztekammern) and Associations of Statutory Health Insurance Physicians (Kassenärztliche Vereinigungen) of different German states. We contacted the chambers and associations of all 16 states of Germany. While some could not answer our request due to high workload or a lack of contact to MAs, the chamber and/or association of 10 states supported our study (i.e., Baden-Wuerttemberg, Rhineland-Palatinate, Thuringia, Hesse, Bremen, Mecklenburg-Hither Pomerania, Saarland, Saxony-Anhalt, Schleswig-Holstein, North Rhine-Westphalia). All channels provided brief information on the topic of the study and a link to the survey. Inclusion criteria for participation were (a) currently working as an MA and (b) being 16 years or older. Participants explicitly declared that they fulfil these obligations by clicking the button that leads to the next page. Further, after giving informed consent, participants answered a questionnaire including the study variables (see ‘Measures’). We refrained from extrinsic remuneration of participants and, instead, drew upon participants’ intrinsic motivation by emphasizing that each participation helps our research that aims at supporting the MA-profession. Data collection ran from September to October 2022.

### Measures

The survey administration and all measures were in German language. At the beginning of the questionnaire we assessed some demographic information. First, we asked for age and gender of the participants, as studies on the comic styles show that these two characteristics relate differently to comic styles as well as to work-outcomes [21, 34]. We also included professional experience (i.e., years working as MA) as this might influence especially competency. Finally, we asked for the current professional area participants work in.

The eight comic styles were assessed with the German version of Ruch et al.’s [20] 48-item self-report questionnaire (6 items per style). Examples for each style are: “I accept the imperfection of human beings and my everyday life often gives me the opportunity to smile benevolently about it.” (*benevolent humour*), “I like to make jests and to be silly.“ (*Fun*), “I have the ability to tell something witty and to the point.” (*Wit*), “I like humour when it aimlessly plays with sense and nonsense.” (*nonsense*), “If I say something that is ironic, there is always someone in my group who understands it, and others who don’t.” (*Irony*), “I caricature my fellow humans’ wrongdoings in a funny way to gently urge them to change.“ (*Satire*), “Biting mockery suits me.” (*Sarcasm*), “In general, human beings and the world are weak and I don’t mind devaluating generally accepted values by cynical remarks” (*cynicism*). The questionnaire is validated in different languages, including German and English [20]. The full set of the German and English items can be found in the open source supplementary material of Ruch et al.’s publication [20]. As the samples of the validation studies of the questionnaire had relatively high educational levels compared to our study’s target group, we made small linguistic adaptations to some items by adding synonyms to complex words (e.g., *critical* to cynical), in order to ensure comprehension. In terms of a pre-test, two MAs independently confirmed the comprehensibility of the items. Participants rated the items on a scale from 1 (strongly disagree) to 7 (strongly agree). Cronbach’s Alpha ranged from .74 (benevolent humour) to .86 (sarcasm) in this sample.

PPF was measured in terms of different facets of work-related (a) well-being and (b) competence. To capture well-being, we chose two constructs. First, we integrated the German version of the 9-item *work engagement scale* by Schaufeli et al. [35] that consists of the three subscales *vigour* (sample item: “At my job, I feel strong and vigorous”,), *dedication* (sample item: “I am enthusiastic about my job”) and *absorption* (sample item: “I am immersed in my work”). Participants rated the items on a scale from 1 (never) to 7 (always). The scale is validated in several languages, including German [36] and reached an internal consistency of 0.94 in our study. Further, we assessed *work satisfaction* with the 5-item scale by Haarhaus [37]. The items (e.g., “All in all, my job is good.”) were rated on a scale from 1 (does not apply at all) to 10 (does fully apply), yielding an alpha of 0.90.

To capture competence, we included three indicators. First, we assessed occupational self-efficacy with Rigotti et al.`s [38] 6-item instrument (e.g., “Whatever comes my way in my job, I can usually handle it.”). The instrument uses a scale from 1 (does not apply at all) to 6 (does fully apply). It is validated in German and several other languages and its items are included in the appendix of Rigotti et al.’s publication [38]. The scale reached an alpha of 0.89 in our study. Second, drawing on Sparr and Sonnentag [39], participants rated the amount of spontaneous positive feedback they receive during one work day by (a) patients, (b) colleagues and (c) supervisors (scale from “not once a day” to “more than four times a day”, see Additional_file_1). As we were interested in the overall feedback, we used the mean of the three ratings for the analyses (alpha = 0.59). Third, we asked the participants if they currently were an MA in a leading position (yes/no). In addition to performing regular medical assistant tasks, leading MAs take charge of a medical practice or clinical section, leading the team and directing its processes. As their responsibilities exceed those of regular MAs, they frequently hold additional qualifications in areas like practice management [40]. Therefore, this item was used as a more objective indicator for MAs’ occupational competence.

### Analyses

For data preparation as well as descriptive and correlational analyses we used SPSS version 25 of the IBM Deutschland GmbH (Ehningen, Germany) and R version 4.2.1 and R studio version 2022.12.0 for regression analyses and the corresponding plots; packages: “GGally” [41] and “broom.helpers” [42]. To test the hypotheses, for the metric facets of PPF partial Pearson correlations (coefficients = *r*) as well as multiple linear hierarchical regression analyses with z-scores (coefficients = *β*) were conducted. Further, for the dichotomous facet *Leading MA*, ANCOVAs as well as logistic hierarchical regressions (odds ratios = OR) were chosen. Coefficients and odds ratios that were statistically significant with *p* < .05 were seen as substantial in this study. For the regression analyses, we also report the respective confidence intervals (CI). Confidence intervals that do not include the specific value (0 for hierarchical regressions, 1 for logistic regression) indicate a significance level of *p* < .05. We avoid categorizing coefficients as weak or strong definitively and only use these terms for comparisons of coefficients. For all analyses, age and professional experience were used as control variables. Investigating the relationships not only individually for each comic style but also in a joint regression model enables to control for the general “sense of humour” factor that all comic styles share. Thus, regression coefficients should refer to the mere style.

All analyses were further conducted with the subsample of MAs working in general medicine (*n* = 263) to address the research question regarding replicability. As assumed, the subsample reported working in significantly smaller practices with smaller teams than the rest of the sample (*p* < .001). However, the descriptive statistics of the comic styles and PPF did not differ significantly between the two subgroups.

## Results

### The study’s sample

While 1427 participants started the survey in the first place, 625 completed it (completion rate = 44%). Participants quitted especially on the first pages of the survey that included either items regarding ones’ current employment or items of the comic styles. Low response rates and high dropout rates are typical risks of anonymous online-surveys – however, in our view, justifiable for the sake of full anonymity of the participants [43]. Further, we excluded 17 participants due to occasional missing values or unrealistic processing times (i.e., reading and answering one item in less than 3 s). The final sample for the analyses was *N* = 608 (see Table 1). The overall number of MAs in Germany was almost 500.000 in 2023 [44]. This would suggest, that our sample comprises about 0.12% of all current German MAs. However, our sample’s composition regarding population characteristics is very similar. Almost all participants were female (98%) and stated German as their only native language (94,1%). Further, most participants had an intermediate school-leaving certificate as highest school level (68%). These demographics are typical of the MA-profession in Germany [4, 44]. Additionally, most participants stated that they were employed in the ambulant sector (91,5%) and more than a third of the participants currently worked in the field of general medicine (43%). These shares are representative for this occupation in Germany [6, 31]. Participants’ age (16–65 years) and professional experience (< 1–53) were normally distributed.

**Table 1:** Sociodemographic characteristics of the total sample of medical assistants (*N* = 608)

Characteristics	Mean (SD)	n (%)
Gender		
female		598 (98.4)
male		10 (1.6)
Age (years)	41.21 (11.12)	
16–36		203 (33.4)
37–46		197 (32.4)
47–65		208 (34.2)
Highest level of education^1^		
Low		35 (5.8)
Intermediate		413 (67.9)
High		160 (26.3)
Other		29 (4.7)
Native language		
German		572 (94.1)
German + other language(s)		16 (2.6)
Other language(s)		20 (3.3)
Professional experience as MA (years)	17.96 (11.42)	
< 1–10		209 (34.4)
11–24		211 (34.7)
25–53		188 (30.9)
Currently leading MA^2^		
Yes		201 (33.1%)
no		407 (66.9%)
Current employer		
Single practice		234 (38.5)
Joint practice		260 (42.8)
Medical care centre		62 (10.2)
Hospital/Clinic		46 (7.6)
Other		6 (1.0)
Current medical field		
General medicine		263 (43.3)
Special field (e.g., paediatrician, orthopaedist)		345 (56.7)
Employment scheme		
Full-time		317 (52.1)
Part-time		291 (47.9)

Note^1^Low = no or lower school-leaving certificate; Intermediate: secondary school-leaving certificate; High: upper secondary school-leaving certificate (i.e., qualification for university entrance or university of applied sciences), ^2^leading MA = In addition to performing regular medical assistant tasks, leading MAs take charge of a medical practice or clinical section, leading the team and directing its processes

### Descriptive statistics of the comic styles and partial correlations between the styles and PPF

Table 2 depicts the descriptive statistics of the comic styles as well as their partial correlations with PPF. Overall, the light styles showed higher means and smaller standard deviations than the dark styles. Further, the styles benevolent humour, fun and wit showed significant positive correlations with most of the facets of PPF (*p* < .05). However, the correlations differed in magnitude depending on the facet. For example, regarding well-being, benevolent humour had the highest positive correlations, while regarding the competency-facets, wit showed the highest correlations (e.g., self-efficacy: *r* = .27, 95% CI [0.20, 0.34]). Nonsense showed coefficients close to zero with most of the facets. Sarcasm and Cynicism correlated only with job satisfaction, however negatively (e.g., sarcasm: *r* = .13, 95% CI [-0.21, -0.05]). Further, the correlations between irony and positive feedback (*r* = .11, 95% CI [0.03, 0.19]) and between satire and work engagement (*r* = .10, 95% CI [0.02, 0.18]) were significant.

**Table 2:** Partial correlations and ANCOVAs of the comic styles and PPF (controlled for age and work experience)

	M^1^	SD		Partial Correlations								F-Values^2^	
				Work Engagement		Job Satisfaction		Occupational Self-Efficacy		Positive Feedback at work		Currently Leading MA	
				Total	GM	Total	GM	Total	GM	Total	GM	Total	GM
Ben.	5.23	0.82	*r*	**0.23**	**0.15**	**0.13**	0.04	**0.22**	**0.26**	**0.13**	**0.17**	0.38	1.73
			*p*	<0.001	0.015	0.002	0.481	<0.001	<0.001	0.002	0.007	0.540	0.190
Fun	4.98	1.07	*r*	**0.19**	**0.20**	0.06	0.07	**0.10**	**0.17**	**0.16**	**0.21**	0.25	0.77
			*p*	<0.001	0.001	0.114	0.239	0.013	0.007	< 0.001	0.001	0.617	0.382
Wit	5.03	0.94	*r*	**0.17**	0.12	0.02	-0.03	**0.27**	**0.25**	**0.16**	**0.18**	**4.91**	0.92
			*p*	<0.001	0.062	0.688	0.628	<0.001	< 0.001	<0.001	0.004	0.027	0.337
Non.	4.71	1.18	*r*	0.05	0.05	-0.07	-0.08	0.06	0.09	0.05	0.11	2.00	0.74
			*p*	0.189	0.457	0.079	0.185	0.134	0.158	0.271	0.084	0.158	0.390
Irony	4.75	1.11	*r*	0.04	0.06	-0.06	-0.10	0.08	0.15	**0.11**	**0.17**	0.63	0.11
			*p*	0.303	0.360	0.179	0.092	0.055	0.017	0.005	0.008	0.428	0.739
Satire	4.16	1.08	*r*	**0.10**	0.12	-0.05	-0.07	0.07	0.09	0.07	**0.16**	0.03	0.51
			*p*	0.016	0.057	0.273	0.268	0.083	0.147	0.079	0.008	0.863	0.476
Sarc.	3.92	1.38	*r*	-0.07	-0.01	**-0.13**	**-0.13**	-0.01	0.045	-0.01	0.03	0.27	1.68
			*p*	0.078	0.869	0.002	0.033	0.873	0.432	0.868	0.681	0.602	0.197
Cyn.	3.62	1.17	*r*	-0.04	-0.02	**-0.15**	**-0.17**	0.02	0.11	0.03	0.07	0.08	0.00
			*p*	0.358	0.772	<0.001	0.005	0.719	0.067	0.517	0.278	0.783	0.980

*Note* Total = Total Sample, *N* = 608, GM = Subsample of MAs working in General Medicine, *n* = 263; ^1^Means and Standard Deviations for overall sample; ^2^ANCOVA: 0 = no leading MA vs. 1 = leading MA; Ben = Benevolent Humour, Non = Nonsense, Sarc. = Sarcasm, Cyn. = Cynicism; *r* = correlation coefficient; coefficients in **bold** are significant with *p* < .05 and seen as substantial in this study. We avoid categorizing coefficients as weak or strong definitively and only use these terms for comparisons of coefficients

### Hierarchical regression analyses of PPF on the comic styles

**Fig. 1:**
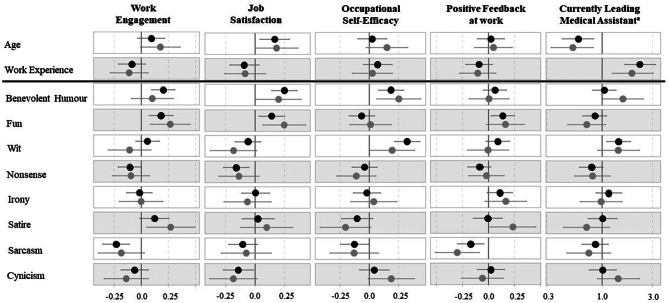
Coefficients of hierarchical regressions of PPF on control variables and the comic styles. *Note* Depicted are the standardised beta-coefficients of hierarchical regressions and the ^a^Odds Ratios of logistic regressions (= dots) including confidence intervals (= horizontal lines next to dots); confidence intervals that do not cross the vertical zero-line indicate that the respective coefficients/odds ratios are significant with *p* < .05. Those coefficients are seen as substantial in this study. We avoid categorizing coefficients as weak or strong definitively and only use these terms for comparisons of coefficients; black dots = Total sample, *N* = 608; grey dots = Subsample of MAs working in General Medicine, *n* = 263

Although the comic styles interrelated moderately (i.e., *r* = .36–0.71), multicollinearity could be statistically excluded (VIF < 10) and thus, regression results are interpretable. Figure 1 depicts coefficient plots of the second step of the hierarchical regressions (see also Additional_file_2). The plots show that the directions of most coefficients are similar to the ones of the partial correlations (e.g., benevolent humour and fun positive vs. sarcasm negative). Yet, some results are different from the correlations. For example, while wit correlated with four of five facets, it predicts only occupational self-efficacy (*β* = 0.32, 95% CI [0.22, 0.43]) and being leading MA (OR = 1.46, 95% CI [1.11, 1.86]) significantly. Further, some coefficients increased or even emerged new. For example, nonsense predicts job satisfaction negatively (*β* = − 0.16, 95% CI [-0.27, -0.05]). Further, while sarcasm shows a significant correlation only with job satisfaction, it predicts three facets negatively in the regression analysis (e.g., work engagement: *β* = 0.23, 95% CI [-0.31, -0.15]).

### Analyses for the subgroup of MAs working in general medicine

In the subsample, the directions of most correlational and regression coefficients are similar to the ones of the total sample (e.g., light styles rather positive vs. sarcasm rather negative). However, in the subsample, there was no difference in wit between leading MAs compared to non-leading assistants. Further, regarding positive feedback almost all comic styles showed numerically higher coefficients in the subgroup, with satire even becoming significant (*r* = .16, 95% CI [0.04, 0.28]). In the regression analysis, benevolent humour and fun still predicted most facets positively. Fun even showed higher coefficients in the subgroup than in the total sample (e.g., with work engagement: *β* = 0.27, 95% CI [0.08, 0.45]). However, some coefficients were of smaller size in the subsample and sometimes not insignificant anymore (e.g., occupational self-efficacy on wit: from *β* = 0.32, 95% CI [0.22, 0.43] to *β* = 0.20, 95% CI [< 0.00, 0.39]). On the other side, some coefficients were only significant in the subsample: satire, for example, predicted positive feedback only in the subgroup (*β* = 0.23, 95% CI [0.01, 0.45]).

## Discussion

### Summary and interpretation of the results and comparison with other research

Medical assistants play a crucial role in the healthcare system, yet they often face resource limitations and difficult working conditions including multitasking and working with patients that are under psychological stress [8, 45]. Humour has been recognized as a potential resource for healthcare professionals that helps to cope with these job challenges, but there is a lack of research on humour styles/comic styles in the healthcare context and how these styles relate to professionals’ well-being and competence (i.e., PPF). We narrowed this gap in the literature: As expected, the comic styles relate differently to facets of PPF [20]. Overall, the styles *benevolent humour* and *fun* show the highest positive relations to most of the facets, while, especially, *sarcasm* relates negatively. Most relationship patterns are also prevalent in the subgroup of MAs working in general medicine. The results not only shed light on the differing adaptivity of MAs’ comic styles but also suggest that the styles relate to MAs’ PPF on different levels: On a subjective emotional level (e.g., how vigorous MAs feel during work), on a subjective cognitive level (e.g., how competent MAs evaluate themselves when facing job challenges) and even on an objective level (e.g., currently being leading MA of one’s team). Altogether, the eight styles explain up to 10% of MAs’ PPF (see Additional_file _2). Considering the number of possible antecedents of well-being and performance at work (e.g., autonomy, social support) [46], this suggests a relatively high relevance of the comic styles for MAs’ PPF at work. Future studies should investigate the incremental value of the styles over regularly investigated antecedents (e.g., job demands and resources) [47]. In general, future studies should focus more on personal resources as these are just as relevant for employees’ well-being and competence as job resources (e.g., co-worker support) [46].

The light styles – especially, benevolent humour – predicting most of the facets of PPF positively, supports Hypothesis 1 as well as the previous literature on the comic styles and well-being [18, 22]. However, the relevance of the individual styles seems to depend on the facet of PPF. The results suggest wit to be particularly important for occupational self-efficacy and being leading MA. This may be because wit means to be able to “quickly read situations” and to “nail non-obvious matters to the point in a funny way” [20]. Witty individuals can adapt their humour to various workplace scenarios (e.g., interactions with patients vs. among colleagues), fostering positive social interactions, or helping humorously alleviating stress and tension and, thereby, contributing to a more positive and supportive workplace atmosphere. Their skill in creating surprising punch lines and making accurate judgments in a funny manner can potentially enhance their problem-solving abilities. This, in turn, may relate to their work-related self-efficacy—or, at least, the perception thereof – and may help them to climb the career ladder [48].

Contrary to Hypothesis 1 and the study of Ruch et al. [18], nonsense humour showed no or even negative relations to PPF – especially, to the well-being-facets. A statistical explanation could be suppressor effects due to other styles (e.g., benevolent humour binding the “positive variance” that explains well-being). A content-related explanation could be that high nonsense, indeed, may be maladaptive for MAs. Studies suggest that people high in nonsense (a) have lower conscientiousness and (b) prefer using humour on their own, avoiding interactions with others [20, 49]. However, conscientious working behaviour (e.g., due to high responsibility for others’ and one’s own health) [50] as well as functioning social relationships (e.g., with colleagues) are both crucial in the medical field. Hence, the playful and cheerful nature of nonsense humour – while entertaining for some – may (if overdone) not align well with the demands and values of the medical profession and indicate a poor person × (work) environment fit, that may even lead to medical errors and a decreased well-being at work [51].

As expected in Hypothesis 2, the styles *irony* and *satire* showed positive relations to at least some indicators of MAs’ PPF. This supports Ruch et al. [18] who found correlations of *r* = .10 with positive affect and life satisfaction. Further, the results suggest that satire might be especially relevant for MAs working in general medicine. As general practitioners are characterised by more regular patients [32], interpersonal relationships might be stronger. Therefore, satire (e.g., making patient aware of unhealthy behaviour in a satirical way) may be more welcomed by others [17], which may lead to more positive (or less negative) feedback, which in turn can increase one’s work engagement [11]. Future research should investigate which comic styles might be particularly adaptive in other medical fields (e.g., in oncology, cynicism may be helpful to cope with severe diagnoses; in urology, funny teasing might be less appropriate due to intimate situations). Further, even benevolent humour might be maladaptive in some situations (e.g., highly frightened patients/relatives in emergency situation) [17]. Again, the ability to “read” a situation and choose an appropriate humorous response may be beneficial in this particular work environment.

Hypothesis 3 posited that sarcasm and cynicism show negative relations to MAs’ PPF. Yet, the results suggest especially sarcasm to be maladaptive. This is contrary to other studies, which found higher correlations for cynicism with well-being [18, 28]. Reason for this could be a mismatch between sarcasm and the MA-role. While cynicism is primarily aimed at criticising people in general, sarcasm aims at hurting others, including enjoying others’ harm/mishap. However, working in healthcare requires understanding and empathy (e.g., for worried patients or one’s own mistakes) [52]. Thus, being sarcastic might lead to disapproval by others and to an emotional distance to one’s work. Cynicism might not be taken as personally by others, or they may even share one’s cynical opinion towards the aim of the cynicism (e.g., unreasonable guidelines of health insurances that render a patient’s treatment impossible) [23]. Correspondingly, the regression results of the subgroup suggest that cynicism might even relate positively to occupational self-efficacy and being leading MA.

To our knowledge, this is the first study that uses the comic style framework to investigate humour styles’ relationships to employees’ outcomes in a certain profession. As the comic style framework is relatively new (from 2018), studies using this framework are generally scarce, but increasing. This restricts the comparison of our results to studies with other professions. Nevertheless, there are some approaches that used other humour style frameworks. For example, studies with other healthcare professions showed that the positive relationship between light humour (i.e., *affiliative* and *self-enhancing humour*) and well-being also applies for nurses and doctors [53, 54]. On the other side, so-called *aggressive humour* – which highly overlaps with the comic style *sarcasm* [55] *–* correlates negatively with well-being. These results confirm our study’s results. Additionally, there are a few studies that investigate the relevance of humour styles in other service professions (e.g., hospitality, sale) as well as professions in the educational sector (i.e., teachers). Overall, these studies also confirm the positive relationships between light humour styles and employee outcomes (e.g., job performance, job embeddedness, low burnout) [56–58] However, they also show that *aggressive humour* in some contexts shows zero or even positive relationships [56, 57]. Further research with different professions is needed, to investigate if the adaptivity of humour styles is occupation-dependent (i.e., which styles are especially helpful in which professions? ).

### Limitations

This study comes with several limitations. First, due to the cross-sectional design, causality cannot be inferred. It might be possible that PPF influences the usage of certain comic styles (e.g., having high job strain may lead to increased usage of dark humour) [59], but also that there are interactions and/or third variables that have an impact. In fact, the literature on resources at work suggests that personal resources (e.g., comic styles) and well-being influence each other mutually, maybe even ending up in a positive upward spiral [60]. Second, although we integrated one objective measure of competence (i.e., being leading MA), all constructs were assessed with self-ratings, which may have caused a common-method bias.

Third, the representativity of our sample is restricted. The study’s sample is relatively small (*n* = 625 vs. *N* = 500.000) and we do not have information on the distribution of participants’ region, where they are currently employed. Nevertheless, the composition regarding other key characteristics (i.e., gender, education, migration background, employment, medical field) is – despite the small sample size – very similar to the MA-population in Germany, which supports a certain representability [4, 6, 31, 44]. Fourth, our study only refers to MAs working in Germany. As studies suggest that the prevalence of comic styles and their relations to demographic variables differ slightly between cultures [61] there are restrictions regarding the generalizability of results to other countries. Nevertheless, as the general patterns (e.g., light styles having higher means than dark styles) are similar over cultures, the main results (e.g., benevolent humour more adaptive than sarcasm) probably still hold for other countries. Still, we particularly encourage cross-cultural research to examine this assumption further. The restriction regarding generalizability also applies for gender. Due to our high share of women, we cannot make a statement about men who work in the MA-profession – though they only represent about 3% of all MAs. As other studies on humour styles show differing means in the styles between men and women (e.g., men show higher dark humour) [20, 21], it would be interesting to examine if also the adaptability of the styles differs between the genders.

## Conclusions

This study provides initial data-based insights into the adaptive and maladaptive comic styles for MAs’ PPF at work. It suggests that fostering benevolent humour and fun would be beneficial, while caution should be exercised regarding sarcasm (at least when shared with others). Although the adaptive styles are already prevalent in the group under investigation (see Table 2), sarcasm appears to be relatively widespread (mean score of 3.93 on a 1–7 scale). As we do not have longitudinal data, it would be interesting to see whether, for example, more sarcastic people are attracted to this profession, or whether this work environment has an impact (or alternative explanations apply). The dark but virtuous style of satire could serve as a more adaptive alternative to sarcasm. For instance, instead of sarcastically commenting on a patient’s demanding behaviour, one can use satire to humorously point out their misbehaviour in order to encourage positive change [17].

Previous attempts to humour trainings have successfully increased humour and well-being among healthcare professionals [62]. However, these trainings primarily focus on developing a general sense of humour without distinguishing between different comic styles [63]. Therefore, this study offers initial insights on how to enhance vocational education and training for MAs by encouraging a benevolent perspective and discouraging a sarcastic outlook towards others and one’s own work. Hence, this could be a starting point for the development of a training program particularly targeting MAs. As the literature on resources and employee outcomes suggests the relationships between humour and PPF to be reciprocal in the long-term (i.e., upward spiral) [11, 60] – training MAs’ light humour styles might improve different facets of MAs PPF, which in turn, help MAs to keep their light humour style, even in the face of adversities. In the long run, this spiral might not only help to maintain MAs’ mental and physical health but also to increase their performance and reduce turnover rates [12–14], which in turn, should supports a functioning primary healthcare system. Future studies with longitudinal and/or experimental designs are needed to examine these long-term and reciprocal relationships. Additionally, studies should differentiate between professions to extend research regarding the occupation-specific adaptivity of different humour styles and, thus, to help employees maintain their PPF in their respective working-contexts.

### Electronic supplementary material

Below is the link to the electronic supplementary material.

Supplementary Material 1

Supplementary Material 2

## Data Availability

The dataset supporting the conclusions of this article is not publicly available due to the authors are still working on them but are available from the corresponding author on reasonable request.

## Abbreviations

MA: Medical Assistant
PPF: Positive psychological functioning
*r*: Correlation coefficient
*β*: Regression coefficient beta
*OR*: Odds ratio of logistic regression
*CI*: Confidence Interval
