# Supplementary material for: Medical assistants’ comic styles and their potential for positive functioning at work: a cross-sectional study including a subgroup analysis
Source: BMC Prim Care. 2024 May 7;25:156. doi: 10.1186/s12875-024-02363-y (PMC11075284; doi:10.1186/s12875-024-02363-y)
Supplement: Supplementary file 1 — Supplementary Material 1 [file 12875_2024_2363_MOESM1_ESM.docx]

**Items measuring Positive Feedback at work (cf. Sparr and Sonnentag, 2008)**

“Please think about a typical workday as a MA: How often do you receive positive feedback (e.g., praise or compliments) about your behaviour or performance at work without having specifically asked for it?

a) …from patients? b) …from your supervisor(s)? c) ...from your colleagues?”

**scale**: not once a day; one to two times a day; three to four times a day; more than four times a day
