# Supplementary material for: Medical assistants’ comic styles and their potential for positive functioning at work: a cross-sectional study including a subgroup analysis
Source: BMC Prim Care. 2024 May 7;25:156. doi: 10.1186/s12875-024-02363-y (PMC11075284; doi:10.1186/s12875-024-02363-y)
Supplement: Supplementary file 2 — Supplementary Material 2 [file 12875_2024_2363_MOESM2_ESM.docx]

**Results of hierarchical regressions of PPF on control variables and the comic styles**

|  |  | **Work Engagement** | |  | **Job Satisfaction** | |  | **Occupational Self-Efficacy** | |  | **Positive Feedback at work** | |  | **Currently Leading MA^a^** | |  |
| --- | --- | --- | --- | --- | --- | --- | --- | --- | --- | --- | --- | --- | --- | --- | --- | --- |
|  |  | Total | GM |  | Total | GM |  | Total | GM |  | Total | GM |  | Total | GM |  |
| *Step 1* | *R*^2^ | *0.00* | *0.01* |  | *0.02* | *0.01* |  | *0.02* | *0.03* |  | *0.00* | *0.00* |  | *0.07* | *0.03* |  |
| Age | *β* /OR | 0.10 | **0.19** |  | **0.20** | **0.20** |  | 0.04 | **0.18** |  | 0.01 | 0.08 |  | **0.64** | **0.65** |  |
|  | CI | [-0.03,0.23] | [0.01,0.37] |  | [0.07,0.33] | [0.02,0.38] |  | [-0.09,0.17] | [0.01,0.36] |  | [-0.12,0.13] | [-0.09,0.26] |  | [0.46,0.88] | [0.42,0.96] |  |
| WE | *β* /OR | -0.05 | -0.10 |  | -0,10 | -0.10 |  | 0.12 | 0.03 |  | -0.06 | -0.09 |  | **2.20** | **1.68** |  |
|  | CI | [-0.18,0.08] | [-0.27,0.08] |  | [-0.29,0.02] | [-0.27,0.08] |  | [-0.01,0.24] | [-0.15,0.20] |  | [-0.18,0.07] | [-0.26,0.09] |  | [1.61,3.08] | [1.13,2.58] |  |
| *Step 2* | *R*^2^ | *0.10* | *0.07* |  | *0.09* | *0.07* |  | *0.12* | *0.11* |  | *0.04* | *0.05* |  | *0.10* | *0.11* |  |
| Age | *β* /OR | 0.09 | 0.18 |  | **0.17** | **0.18** |  | 0.03 | 0.15 |  | 0.02 | 0.05 |  | **0.59** | **0.53** |  |
|  | CI | [-0.03,0.22] | [-0.01,0.36] |  | [0.04,0.29] | [0.00,0.36] |  | [-0.10,0.15] | [-0.03,0.33] |  | [-0.11,0.15] | [-0.13,0.23] |  | [0.24,0.83] | [0.33,0.82] |  |
| WE | *β* /OR | -0,09 | -0.11 |  | -0.09 | -0,08 |  | 0.07 | 0.03 |  | -0.09 | -0.10 |  | **2.27** | **1.91** |  |
|  | CI | [-0.21,0.04] | [-0.29,0.06] |  | [-0.21,0.03] | [-0.26,0.09] |  | [-0.05,0.16] | [-0.14,0.20] |  | [-0.22,0.04] | [-0.28,0.07] |  | [1.64,3.20] | [1.25,3.04] |  |
| Ben. | *β* /OR | **0.20** | 0.10 |  | **0.25** | **0.20** |  | **0.19** | **0.25** |  | 0.06 | 0.00 |  | 1.05 | **1.57** |  |
|  | CI | [0.10,0.31] | [-0.09,0.29] |  | [0.14,0.36] | [0.01,0.39] |  | [0.08,0.29] | [0.07,0.44] |  | [-0.05,0.17] | [-0.19,0.20] |  | [0.82,1.35] | [1.02,2.46] |  |
| Fun | *β* /OR | **0.18** | **0.27** |  | **0.14** | **0.25** |  | -0.06 | 0.01 |  | **0.14** | 0.16 |  | 0.85 | 0.72 |  |
|  | CI | [0.07,0.29] | [0.08,0.45] |  | [0.03,0.25] | [0.07,0.43] |  | [-0.17,0.05] | [-0.17,0.19] |  | [0.02,0.25] | [-0.02,0.34] |  | [0.66,1.10] | [0.47,1.08] |  |
| Wit | *β* /OR | 0.06 | -0.11 |  | -0.06 | -0.18 |  | **0.32** | **0.20** |  | 0.09 | 0.00 |  | **1.43** | 1.42 |  |
|  | CI | [-0.05,0.17] | [-0.31,0.09] |  | [-0.17,0.05] | [-0.38,0.01] |  | [0.22,0.43] | [0.00,0.39] |  | [-0.02,0.20] | [-0.20,0.20] |  | [1.11,1.86] | [0.91,2.25] |  |
| Non. | *β* /OR | -0.11 | -0.10 |  | **-0.16** | -0.14 |  | -0.04 | -0.11 |  | -0.09 | -0.02 |  | 0.79 | 0.81 |  |
|  | CI | [-0.21,0.00] | [-0.27,0.08] |  | [-0.27,-0.05] | [-0.31,0.03] |  | [-0.15,0.07] | [-0.28,0.06] |  | [-0.20,0.02] | [-0.19,0.15] |  | [0.61,1.02] | [0.55,1.19] |  |
| Irony | *β* /OR | -0.02 | 0.00 |  | 0.00 | -0.07 |  | -0.02 | 0.04 |  | 0.11 | 0.17 |  | 1.16 | 0.98 |  |
|  | CI | [-0.14,0.10] | [-0.20,0.20] |  | [-0.11,0.12] | [-0.27,0.14] |  | [-0.14,0.10] | [-0.16,0.23] |  | [-0.01,0.23] | [-0.03,0.37] |  | [0.88,1.53] | [0.62,1.54] |  |
| Satire | *β* /OR | 0.12 | **0.27** |  | 0.03 | 0.10 |  | -0.10 | -0.20 |  | -0.01 | **0.23** |  | 1.01 | 0.71 |  |
|  | CI | [-0.01,0.25] | [0.05,0.49] |  | [-0.11,0.16] | [-0.12,0.32] |  | [-0.24,0.03] | [-0.42,0.01] |  | [-0.15,0.13] | [0.01,0.45] |  | [0.74,1.38] | [0.43,1.17] |  |
| Sarc. | *β* /OR | **-0.23** | -0.18 |  | -0.10 | -0.07 |  | **-0.13** | -0.13 |  | **-0.17** | **-0.30** |  | 0.86 | 0.75 |  |
|  | CI | [-0.35,-0.11] | [-0.40,0.03] |  | [-0.22,0.02] | [-0.29,0.14] |  | [-0.25,0.00] | [-0.34,0.08] |  | [-0.30,-0.04] | [-0.51,-0.09] |  | [0.65,0,1.14] | [0.46,1.20] |  |
| Cyn. | *β* /OR | -0.06 | -0.14 |  | **-0.14** | -0.18 |  | 0.04 | 0.19 |  | 0.02 | -0.06 |  | 1.01 | 1.42 |  |
|  | CI | [-0.19,0.06] | [-0.34,0.06] |  | [-0.27,-0.01] | [-0.39,0.02] |  | [-0.08,0.17] | [-0.01,0.39] |  | [-0.11,0.15] | [-0.26,0.14] |  | [0.75,1.36] | [0.90,2.26] |  |

Note: Total = Total Sample, *N* = 608, GM = Subsample of MAs working in General Medicine, *n* = 263; **^a^**Odds Ratios of logistic regressions (0 = no leading MA vs. 1 = leading MA); Ben. = Benevolent Humour, Non. = Nonsense, Sarc. = Sarcasm, Cyn. = Cynicism; *β* = beta coefficient; OR = Odds Ratio; coefficients and odds ratios in bold are significant with *p* < .05 and seen as substantial in this study. We avoid categorizing coefficients as weak or strong definitively and only use these terms for comparisons of coefficients; *R*^2^ = Adjusted R^2^; CI = 95%-Confidence Interval: Intervals that do not cross the respective threshold (i.e., 0 for hierarchical regressions; 1 for logistic regression) support a significance of *p* <.05
